# NMDA Receptor Expression in the Thalamus of the Stargazer Model of Absence Epilepsy

**DOI:** 10.1038/srep42926

**Published:** 2017-02-21

**Authors:** Z. Barad, D. R. Grattan, B. Leitch

**Affiliations:** 1 Department of Anatomy, Otago School of Biomedical Sciences, University of Otago, Dunedin, New Zealand; 2 Brain Health Research Centre, University of Otago, Dunedin, New Zealand; 3 Centre for Neuroendocrinology, Dunedin, New Zealand

## Abstract

In the stargazer mouse model of absence epilepsy, altered corticothalamic excitation of reticular thalamic nucleus (RTN) neurons has been suggested to contribute to abnormal synchronicity in the corticothalamic-thalamocortical circuit, leading to spike-wave discharges, the hallmark of absence seizures. AMPA receptor expression and function are decreased in stargazer RTN, due to a mutation of AMPAR auxiliary subunit stargazin. It is unresolved and debated, however, if decreased excitation of RTN is compatible with epileptogenesis. We tested the hypothesis that relative NMDAR expression may be increased in RTN and/or thalamic synapses in stargazers using Western blot on dissected thalamic nuclei and biochemically isolated synapses, as well as immunogold cytochemistry in RTN. Expression of main NMDAR subunits was variable in stargazer RTN and relay thalamus; however, mean expression values were not statistically significantly different compared to controls. Furthermore, no systematic changes in synaptic NMDAR levels could be detected in stargazer thalamus. In contrast, AMPAR subunits were markedly decreased in both nucleus-specific and synaptic preparations. Thus, defective AMPAR trafficking in stargazer thalamus does not appear to lead to a ubiquitous compensatory increase in total and synaptic NMDAR expression, suggesting that elevated NMDAR function is not mediated by changes in protein expression in stargazer mice.

Epilepsy is one of the most prevalent chronic neurological disorders, affecting approximately 65 million people worldwide^1^, one third of whom are unresponsive to currently available anti-epileptic medication^2^. Moreover, paediatric epilepsies are particularly common and can have a profound effect on the cognitive development and psychological wellbeing of affected children^3^. Absence seizures are non-convulsive, genetic, generalized seizures that appear in several forms of paediatric and adult epilepsies, with characteristic 3–4 Hz spike-and-wave discharges (SWDs) in EEG accompanied by unresponsiveness and brief loss of consciousness in patients. Children with absence seizures often experience adverse behavioural, psychiatric, and cognitive difficulties, such as attentional problems, anxiety, depression, and reductions in learning and memory^4^,^5^,^6^. This, combined with side effects associated with anti-absence drugs as well as the high rate of patients that don’t respond to available medication, warrant studies examining mechanisms underlying absence seizure generation and propagation. Several rodent models that display absence-like seizures facilitate the understanding of these underlying mechanisms.

Although activation of widespread brain areas can be detected during discharges^7^, SWDs primarily arise from abnormal corticothalamic hypersynchrony. While the precise initiation site of SWDs had been a matter of debate^8^, the currently prevailing cortical focus theory suggests that discharges are initiated in the cortex^9^,^10^. However, global, bilaterally generalized SWDs depend on an intact corticothalamic-thalamocortical (CT-TC) circuit, comprising interconnected cortical and thalamic areas^11^,^12^,^13^. Thalamic lesions, depending on the size and location, can either abolish or aggravate genetically determined SWDs in a rat absence model^14^. In particular, corticothalamic projections to the ventral posterior (VP) relay thalamus, reciprocal thalamocortical projections to the somatosensory cortex, and their collaterals to the parvalbumin (PV)-positive inhibitory neuron-containing reticular thalamic nucleus (RTN) delineate structures implicated to be involved in absence seizure generalization and maintenance^8^,^15^. Imbalance in excitatory/inhibitory synaptic transmission may predispose the circuit to altered synchrony, and ultimately SWDs. The RTN is a key circuit component, intermediate between the thalamic and cortical nodes, that exerts extensive inhibitory control on relay thalamic nuclei upon excitatory CT and TC input, and, therefore, critically determines the synchronicity of circuit oscillations. The cortical input to RTN appears to be of critical importance; in the Gria4^−/−^ mouse model^16^, for instance, a decrease in AMPA receptor function at corticothalamic synapses on RTN neurons leads to SWDs. The loss of Gria4-containing AMPARs at corticothalamic (CT)-RTN synapses results in decreased feed-forward inhibition of relay thalamus, but reveals a new pathway for network hyperexcitability^17^, as the weakened inhibition of the relay nuclei via the CT-RTN-VP route in effect leads to increased direct excitation of VP neurons via corticothalamic projections. This work thus demonstrated that reduction in excitatory AMPA receptors can, paradoxically, result in hyperexcitatory oscillations by shifting the excitatory-inhibitory balance at the network level when the reduction in AMPARs is specific to CT-RTN synapses^17^.

The mouse strain stargazer shows 6–9 Hz SWDs, electrical activity that resembles human absence seizures, and is one of the widely used models for absence epilepsy^18^. The recessive mutation of the AMPA receptor auxiliary protein stargazin (or γ-2)^18^, causes impaired AMPA receptor trafficking^19^,^20^, and pharmacology^21^,^22^,^23^ in several brain regions in stargazers. Stargazin belongs to the family of transmembrane AMPA receptor proteins (TARPs)^24^, members of which may show complementary distribution in the CNS. Stargazin, however, is the predominant TARP in the RTN, and hence, AMPARs are dramatically reduced in stargazer RTN and particularly CT-RTN synapses^25^, also affecting AMPAR-mediated currents^26^. It had been proposed that the marked decrease in AMPAR expression and function at CT-RTN synapses could diminish the CT-RTN-VP feed-forward inhibition, leading to elevated direct CT-VP excitation and network hypersynchrony in stargazers, analogous to the Gria4^−/−^ model^25^,^26^. Yet, functional evidence suggests that improperly trafficked AMPA receptors may remain at perisynaptic locations in stargazer RTN and contribute to altered AMPAR-mediated neurotransmission, which is reduced in amplitude but prolonged in duration^27^. Moreover, Lacey *et al*. reported a compensatory increase in NMDAR-mediated spontaneous and evoked excitatory postsynaptic currents (EPSCs) when recording from stargazer RTN^27^. Overall, the net excitatory charge in stargazer RTN was comparable to controls attributable to the combined effect of the prolonged AMPAR component and augmented NMDAR-mediated currents. The findings also led the authors to argue that the increased NMDAR-mediated RTN excitability is responsible for circuit SWD generation.

The elevated NMDAR-currents could result from the concomitant increase in NMDAR activation in response to glutamate following decreased expression and function of synaptic AMPARs. Alternatively, as suggested, total or synaptic NMDAR expression may be upregulated in stargazer RTN^27^ through compensatory mechanisms. Notably, while the electrophysiological findings pointed to a potential elevation in NMDA receptor numbers in stargazers, they also showed the lack of change in receptor properties, i.e. receptor composition relative to controls^27^. Although NMDARs are not directly associated with stargazin or other members of the TARP family, there have been reports to show that NMDAR expression^19^ and function^28^ may be indirectly affected in stargazers. Specifically, NR1 subunit labelling was increased with post-embedding immunogold cytochemistry at stargazer cerebellar granule cell (GC) synapses that were devoid of the GluA2/3 subunits of AMPARs^19^. Thereby, we hypothesized that NMDAR protein expression may be elevated in the stargazer RTN or at thalamic synapses. The aim of this study was to test this hypothesis by quantifying NMDA receptor expression in the stargazer thalamus in comparison to control littermates. We used Western blot for comparative analysis of NMDAR subunits in dissected RTN and VP nuclei to determine if there are region specific changes in stargazers; furthermore, Western blot was used to examine AMPA- and NMDAR expression in biochemically isolated, pooled thalamic synapses in stargazers. Expression of the obligatory NR1 subunit was probed to test if total NMDAR expression is altered, and expression of NR2A and NR2B subunits to see if NR2A- and/or NR2B-containing subtypes are specifically altered. Lastly, in view of its pivotal role and specific impairment in stargazer mice, NMDAR total protein expression was further explored at CT-RTN synapses using post-embedding immunogold cytochemistry for comparative analysis of NR1 immunogold particles associated with single postsynaptic densities (PSD) of presumptive CT synapses in control and stargazer RTN single sections. Protein expression of AMPAR subunit GluA4 was analysed in parallel to demonstrate region specificity and reproducibility of previous findings in stargazers.

## Results

**Figure 1:**
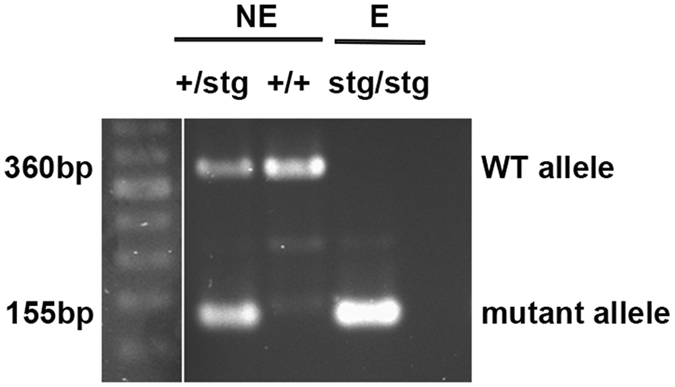
Colony verification using genotyping for the stargazin gene. Representative micrograph indicating the WT (360 bp) and mutant (155 bp) stargazin allele in non-epileptic (NE) control mice (+/stg, +/+) and epileptic (E) stargazer mice (stg/stg).

Adult male mice (8–12 weeks) used in all experiments, derived from stargazer breeding colony, were routinely genotyped to verify the genetic background. Epileptic (E) stargazer mice (stg/stg) were processed parallel with control non-epileptic (NE: +/stg, +/+) littermates (Fig. 1).

### Total NMDAR protein expression is not elevated in RTN and VP of stargazers

**Figure 2:**
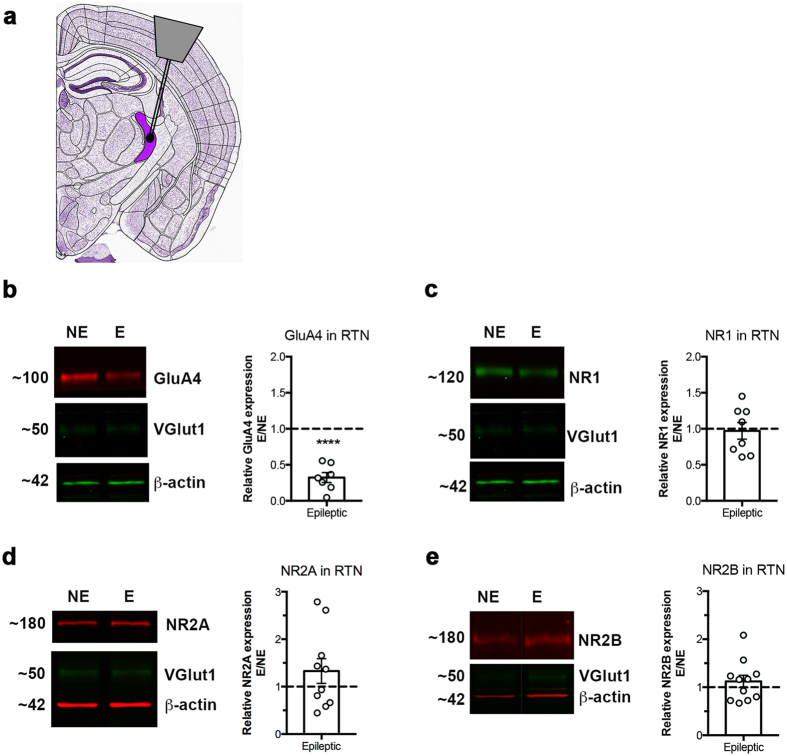
Western blot analysis of AMPA- and NMDA-type excitatory receptor subunits in the RTN. (**a**) Schematic diagram indicating the position of RTN on a coronal mouse brain slice. Modified from Allen Brain Atlas. (http://atlas.brain-map.org/#atlas=1&plate=100960236&structure=549&x=5280&y=3744.0000697544647&zoom=-3&resolution=11.97&z=6). Relative expression of AMPAR subunit GluA4 (**b**), and NMDAR subunits NR1 (**c**), NR2A (**d**), NR2B (**e**) in stargazer RTN; left panel: representative cropped WB images of protein of interest, as well as VGlut1, and β-actin in NE (non-epileptic) and E (epileptic) RTN, right panel: scatter-column combination chart depicting relative epileptic expression of GluA4 (n = 8 NE, n = 7 E; ****p ≤ 0.0001), NR1 (n = 12 NE, n = 8 E; p > 0.05), NR2A (n = 12 NE, n = 10 E; p > 0.05) and NR2B (n = 14 NE, n = 11 E; p > 0.05).

**Figure 3:**
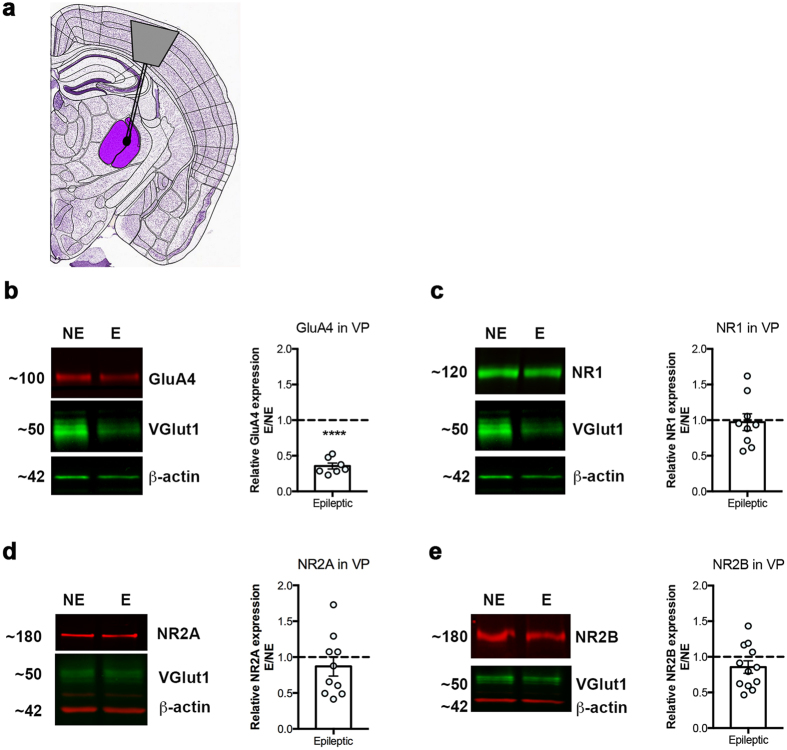
Western blot analysis of AMPA- and NMDA-type excitatory receptor subunits in the VP. (**a**) Schematic diagram indicating the position of VP on a coronal mouse brain slice. Modified from Allen Brain Atlas. (http://atlas.brain-map.org/#atlas=1&plate=100960236&structure=549&x=5280&y=3744.0000697544647&zoom=-3&resolution=11.97&z=6). Relative expression of AMPAR subunit GluA4 (**b**), and NMDAR subunits NR1 (**c**), NR2A (**d**), NR2B (**e**) in stargazer VP; left panel: representative cropped WB images of protein of interest, as well as VGlut1, and β-actin in NE (non-epileptic) and E (epileptic) RTN, right panel: scatter-column combination chart depicting relative epileptic expression of GluA4 (n = 8 NE, n = 7 E; ****p ≤ 0.0001), NR1 (n = 11 NE, n = 9 E; p > 0.05), NR2A (n = 12 NE, n = 10 E; p > 0.05) and NR2B (n = 13 NE, n = 12 E; p > 0.05).

We used Western blot to assess relative AMPA- and NMDAR protein expression in stargazer RTN (Fig. 2) and VP (Fig. 3). RTN somatosensory sectors were dissected from frozen coronal brain sections (Fig. 2a). In parallel, underlying VP nuclei were removed (Fig. 3a) for comparative analysis. Dissection was carried out with the help of anatomical landmarks. Regional specificity was subsequently validated using VGlut1 and GluA4 protein expression. We only analysed RTN samples with low VGlut1-high GluA4 protein expression, as opposed to medium/high VGlut1 and medium GluA4 expression typical of VP, in line with established data^27^,^29^,^30^,^31^. Interestingly, VGlut1 was downregulated in the epileptic VP, which corresponds with the decreased frequency of miniature excitatory postsynaptic currents (mEPSCs) in adult stargazer VP shown before^26^. GluA4 served not only as a regional marker, but also a positive control for stargazers, as total GluA4 protein expression was demonstrated to be reduced in both regions in these mice^25^. GluA4 was detected at 100 kDa, corresponding to the full GluA4 subunit. As expected, intensity of the GluA4-specific band was downregulated in both RTN (68% decrease, n = 8 NE control and n = 7 E stargazer mice; p < 0.0001, one sample t test), and VP (65% decrease, n = 8 NE control and n = 7 E stargazer; p < 0.0001, one sample t test) of stargazers in comparison to non-epileptic controls (Figs 2b and 3b). To compare total NMDAR expression in stargazers and controls, protein expression of the obligatory subunit NR1 was quantified using an antibody that recognizes all NR1 isoforms. The NR1-specific band was detected at 120 kDa. In contrast to GluA4, there was no statistically significant difference in NR1 mean expression between NE and E in either RTN (Fig. 2c) or VP (Fig. 3c) (n = 12 NE, n = 8 E for RTN and n = 11 NE, n = 9 E for VP; p > 0.05, one sample t test).

### NR2A- and NR2B-containing NMDAR subtypes are unaltered in stargazer RTN and VP

In addition to total NMDA receptor levels, reflected by NR1, expression of NR2A- or NR2B-containing subtypes was examined in the stargazer RTN or VP. In line with the literature, NR2A (Figs 2d and 3d) and NR2B (Figs 2e and 3e) were present in both regions and expression was greater in VP compared to RTN^32^,^33^. Mean expression of these subunits was not significantly altered in either region in epileptic stargazers relative to controls (NR2A: n = 12 NE, n = 10 E in RTN and n = 12 NE, n = 10 E in VP; NR2B: n = 14 NE, n = 11 E in RTN and n = 13 NE, n = 12 E in VP; all p > 0.05, one sample t test), based on intensity levels of the 180 kDa bands corresponding to full length NR2A and NR2B subunits, respectively. Heteroscedasticity was tested using Huber-White robust error analysis, which showed no evidence of unequal variability. No statistical difference between the mean protein expression values of all three NR subunits could be detected.

### Decreased AMPAR protein expression at thalamic synapses is not accompanied by permanent upregulation of NMDARs

Although total NMDAR protein expression was not affected in the stargazer RTN, we hypothesised that impaired AMPAR trafficking may impact NMDAR membrane distribution, in particular synaptic NMDAR protein levels in stargazers relative to controls. Therefore, we sought to examine synaptic NMDAR protein expression in relation to AMPARs in stargazers and controls. To this end, biochemical plasma membrane fractionation of thalamic biopsy samples was utilized first. While the technique did not allow nucleus-specific probing of receptor distribution, we reasoned that it could reveal large-scale or ubiquitous changes compensatory to AMPAR reductions.

**Figure 4:**
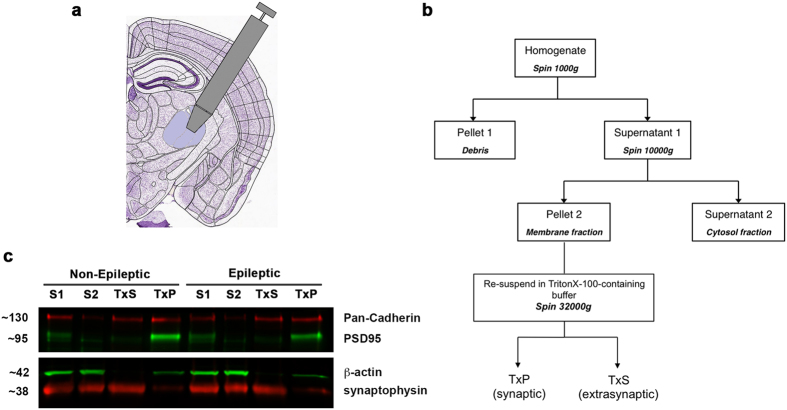
Biochemical fractionation and WB procedure. (**a**) Schematic diagram of a coronal mouse brain section indicating the region removed using biopsy punch pen. Modified from Allen Brain Atlas (http://atlas.brain-map.org/#atlas=1&plate=100960236&structure=549&x=5280&y=3744.0000697544647&zoom=3&resolution=11.97&z=5). (**b**) Flow chart to illustrate fractionation methodology. TxP = Triton X-100 insoluble synaptic fraction (pellet of last spin), TxS = Trinton X-100 soluble extrasynaptic membrane fraction (supernatant of last spin). (**c**) Representative WB composite images showing bands corresponding to markers, Pan-Cadherin and PSD-95 in different fractions. S1 = supernatant 1 - total lysate; S2 = supernatant 2 - cytosol; TxS = Triton X-100 soluble supernatant – extrasynaptic membrane; TxP = Triton X-100 insoluble pellet – synaptic membrane.

Tissue punches 1 mm in diameter containing both the RTN and VP regions were taken for this study (Fig. 4a), to achieve sufficient protein yield from biochemically isolated synaptic membranes for WB analysis. Following isolation of cell membranes, the differential solubility of membrane compartments in non-ionic detergents^34^,^35^,^36^ was utilised to separate non-ionic detergent insoluble synaptic and detergent soluble extrasynaptic fractions (Fig. 4b). The methodology was validated by enriched expression of Pan-Cadherin in both membrane fractions (extrasynaptic: TxS; synaptic: TxP), as well as the TxP fraction-specific enrichment of the bona fide PSD protein PSD95 (Fig. 4c). Expression of receptor proteins was normalized to Pan-Cadherin, as neuronal cadherin is not associated with TARPs^24^, and its expression was not affected in stargazers.

**Figure 5:**
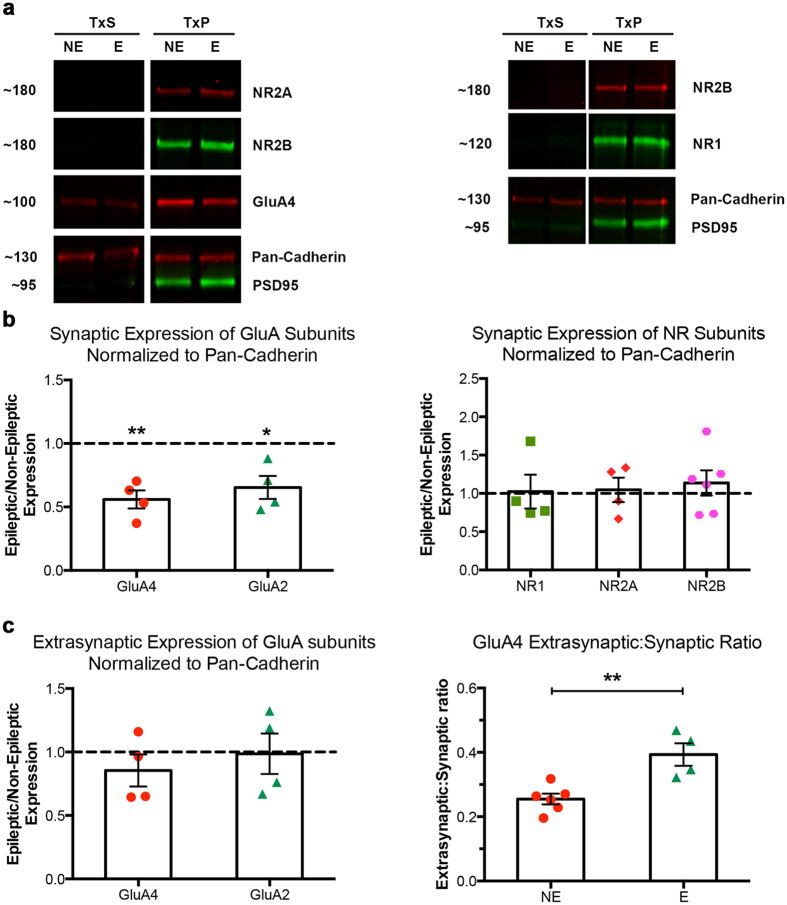
Relative synaptic and extrasynaptic GluA4, NR1, NR2A, and NR2B subunit levels in epileptic stargazer thalamus. (**a**) Representative composite images of cropped GluA4, NR1, NR2A, and NR2B immunoblots with markers Pan-Cadherin and PSD-95. (**b**) Scatter-column combination charts depicting the expression of GluA4 and GluA2, as well as NR1, NR2A, and NR2B subunits in the synaptic membrane fraction of E (epileptic, n = 4 from 4 litters) stargazer thalamus relative to NE (non-epileptic, n = 6 from 4 litters) controls after normalization to Pan-Cadherin (*p ≤ 0.05, ** ≤ 0.01). (**c**) Scatter-column combination charts depicting the expression of GluA4 and GluA2 subunits in the extrasynaptic membrane fraction of E (epileptic, n = 4 from 4 litters) stargazer thalamus relative to NE (non-epileptic, n = 6 from 4 litters) controls after normalization to Pan-Cadherin (p > 0.05). Scatter-column combination charts depicting extrasynaptic to synaptic GluA subunit ratios in NE controls and E stargazers (** ≤ 0.01, *p ≤ 0.05).

Our previous work demonstrated that AMPA receptors are dramatically reduced at specific synapses in RTN in stargazers, but the effect is less pronounced and not significant in VP synapses^25^. Hence, first synaptic AMPAR levels were quantified in the biochemically-isolated membrane fractions. GluA4 (Fig. 5a) and GluA2 subunits were greatly enriched in the synaptic fraction, while some AMPAR subunit protein expression was also detected in the extrasynaptic fraction (Fig. 5a). Relative GluA4 protein levels in stargazer preparations were altered to varying degrees. TxP-specific GluA4 expression was reduced by around 45% (p < 0.01 in four separate experiments; Fig. 5b), whereas extrasynaptic GluA4 levels were not significantly altered in stargazers compared to controls (Fig. 5c), resulting in significantly higher extrasynaptic to synaptic AMPAR expression ratio in epileptic stargazer mice compared to controls (Fig. 5c). TxP-specific GluA2 protein expression was similarly decreased by around 30% in stargazers (p < 0.05 in four separate experiments; Fig. 5b).

In contrast to AMPAR subunits, NR1, NR2A, and NR2B mean synaptic expression values showed no statistically significant difference in epileptic stargazer thalamic synapses, when compared to non-epileptic controls (Fig. 5a,b). In particular, expression of the compulsory subunit NR1, reflecting the total amount of functional NMDARs, was variable in epileptic synaptic fractions relative to controls, but statistical analysis showed no significant change (p > 0.05, three separate experiments, Fig. 5b). Similarly, no systemic changes in synaptic proportions of NR2A- and/or NR2B-subtypes could be detected in the epileptic thalamus. Mean epileptic expression values relative to non-epileptic expression were not significantly different based on one sample t test performed after normalization (p > 0.05 both NR2A, four separate experiments, and NR2B, six separate experiments, Fig. 5b). NR subunit proteins in the extrasynaptic fraction (TxS) were below detectable levels in the biochemical preparations, preventing systematic quantitative analysis of extrasynaptic NMDA receptor expression.

### Loss of synaptic AMPAR-GluA4 expression does not result in increased total NMDAR protein expression at specific synapses in stargazer RTN

**Figure 6:**
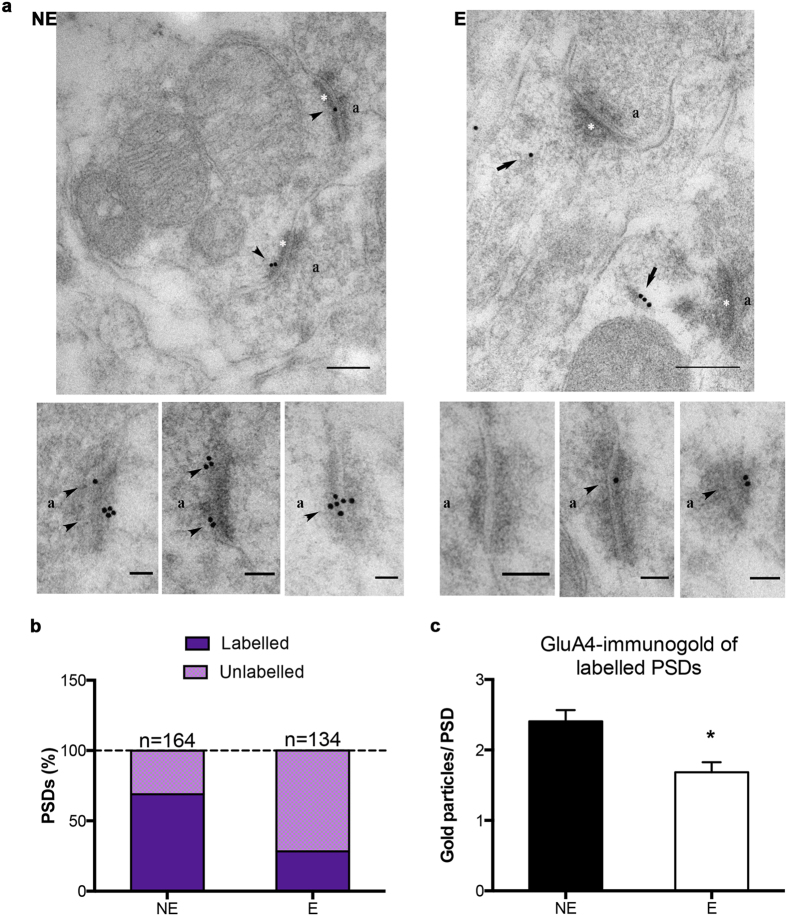
Postembedding immunogold cytochemistry of AMPAR subunit GluA4 in the RTN. (**a**) Sample electron micrographs from NE (non-epileptic) and E (epileptic) RTN sections illustrating GluA4-immunogold particles (15 nm gold, arrow and arrowhead) in NE and E sections. Synaptic targeting and/or anchorage of GluA4 is deficient in epileptic RTN, reflected by GluA4-immunogold particles associated with presumptive intracellular vesicles and/or extrasynaptic membrane, indicated by arrows on the representative E photomicrograph. In contrast, arrowheads indicate PSD-associated GluA4 particles. White asterisks: PSDs, a: presynaptic terminal. Scale bars: 200 nm for large field size, 100 nm for smaller field size images. (**b**) Stacked bars depicting the percentage of GluA4 labelled and unlabelled PSDs in NE and E RTN sections (n = 164 NE and n = 134 E PSDs). (**c**) Bar graphs showing the mean (±SEM) GluA4-gold particles of labelled PSDs in NE and E RTN (*p ≤ 0.05).

**Figure 7:**
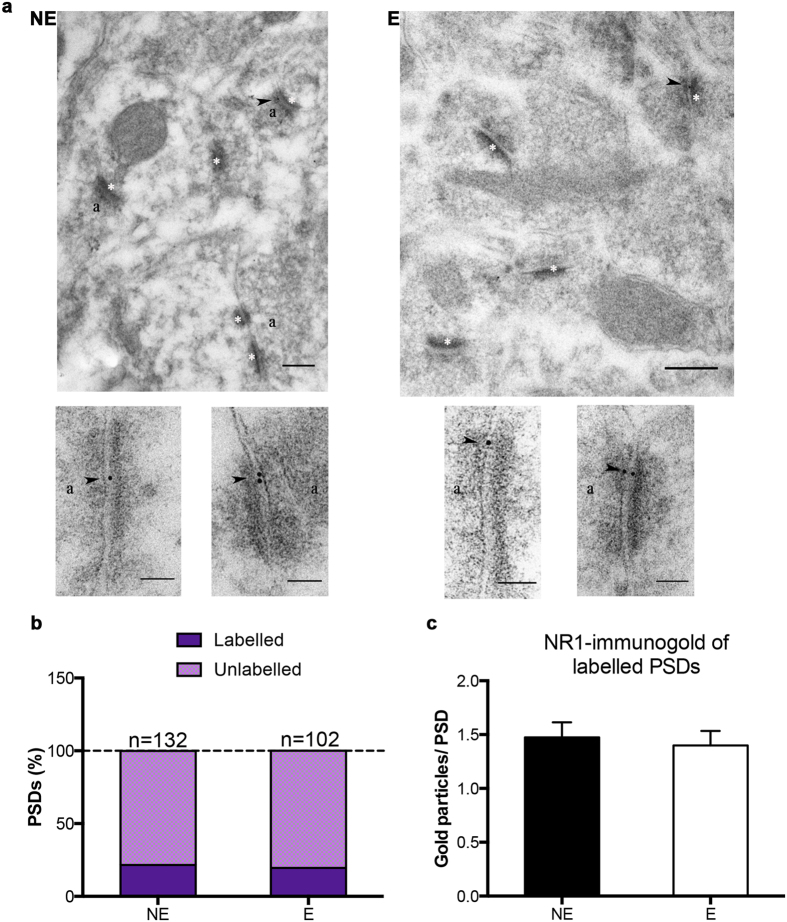
Postembedding immunogold cytochemistry of NMDAR subunit NR1 in the RTN. (**a**) Sample electron micrographs from NE (non-epileptic) and E (epileptic) RTN sections illustrating NR1-immunogold (10 nm gold, arrowhead) particles in NE and E sections. White asterisks: PSDs, a: presynaptic terminal. Scale bars: 200 nm for large field size, 100 nm for smaller field size images. (**b**) Stacked bars depicting the percentage of NR1 labelled and unlabelled PSDs in NE and E RTN sections (n = 132 NE and n = 102 E PSDs). (**c**) Bar graphs showing the mean (±SEM) NR1-gold particles of labelled PSDs in NE and E RTN (p > 0.05).

Findings of the fractionation experiments suggested that even a substantial loss of synaptic AMPARs is not ubiquitously associated with increased NMDAR levels. In order to evaluate synaptic NMDAR expression at the principal CT-RTN synapse further comparative post-embedding immunogold electron microscopy was undertaken in stargazer (E) and control (NE) mice. Ultrathin sections from Lowicryl HM-20-embedded RTN blocks were collected and immunolabelled for GluA4 and NR1 subunits (Figs 6a and 7a). Immunogold particles representing GluA4 and NR1 subunits within 30 nm of asymmetric single PSD profiles, characteristic of CT-RTN synapses, were quantified in single sections of NE-E littermate pairs. Gold particles were generally associated with membrane structures, and labelling was absent from mitochondria, nuclear structures, and blood vessels. Omission controls resulted in negligible labelling.

Immunolabelling for AMPAR-GluA4 (Fig. 6a) confirmed previous findings reflective of AMPAR trafficking defects at the stargazer CT-RTN synapse, including a dramatic decrease in the percentage of GluA4-labelled PSDs (68.90% of a total of 164 PSDs in three NE mice, 28.36% of a total of 134 PSDs in three E mice; Fig. 6b), and significant reductions in the calculated mean number of gold particles per labelled PSDs (2.405 ± 0.1611 in NE, 1.684 ± 0.1416 in E; *p < 0.05; Fig. 6c) in stargazers compared to control littermates. In contrast, NR1 labelling at densities on ultrathin sections from the same RTN blocks was low, but comparable in stargazer and control mice (Fig. 7a). The percentage of labelled PSDs was similar in both groups (21.590% of a total of 132 PSDs from three NE mice, 19.610% of a total of 102 PSDs in three E mice; Fig. 7b), and the calculated mean number of gold particles per labelled PSDs was unchanged in stargazer RTN sections in comparison to controls (1.474 ± 0.1404 in NE, 1.400 ± 0.1338 in E; p > 0.05; Fig. 7c).

## Discussion

Changes to excitatory neurotransmission at specific neuronal populations could render neural circuits prone to epileptiform discharges by resulting in excitatory/inhibitory imbalance. Recent studies indicated that NMDAR function is increased at cortical inhibitory interneurons^37^, and on RTN neurons^27^ in stargazer mice. Our results suggest, however, that the enhanced function in the stargazer RTN is not underpinned by a ubiquitous, permanent increase in NMDAR protein expression. Furthermore, the findings also indicate that the augmented currents are not likely to be driven by synapse-specific permanent increases in protein expression. No constitutive compensatory changes could be detected in NMDAR protein levels in spite of the dramatic reduction in tissue and synaptic GluA4 protein expression, suggesting that NMDAR subunit translation is not increased in stargazers. The lack of compensatory change at the CT-RTN synapse, in particular, could indicate that the impaired AMPAR-mediated corticothalamic excitation of stargazer RTN neurons is not reversed by NMDARs, ensuing feed-forward disinhibition of the relay thalamus in stargazers, in line with previous arguments^25^,^26^. Further research is needed to consolidate these findings, however, and to identify the cellular mechanisms underpinning the elevated NMDAR function in stargazers. Notably, the variability in protein expression could reflect posttranslational modification, or transient changes in synaptic NMDAR incorporation in stargazers, which could contribute to altered RTN neuron excitability. Furthermore, the variability may reflect dynamic changes in neuronal firing mode or electrical activity.

In general, heightened excitability of neural circuits is thought to underlie epileptiform activity. There is mounting evidence to suggest that, in addition to the direct enhanced excitation of principal neurons, decreased synaptic excitation and recruitment of inhibitory neurons could also lead to excitatory/inhibitory imbalance, due to the subsequent weakening of their inhibitory output, and this increased epileptic sensitivity can be rescued by optogenetic activation of inhibitory interneurons^38^,^39^,^40^,^41^. Consistent with this are findings from the Gria4^−/−^ mouse absence model, where impaired AMPAR-mediated corticothalamic excitation of RTN neurons was associated with elevated direct corticothalamic excitation of relay neurons, presumably due to reduced CT-RTN-VP feed-forward inhibition^17^. Corresponding findings of diminished AMPAR function^26^ and expression, in particular at CT-RTN synapses^25^ and somatosensory PV^+^ inhibitory interneurons^28^, suggested the existence of identical circuit mechanisms in the stargazer absence model. Nonetheless, the role of RTN excitability in absence epilepsy circuit mechanisms is controversial as its inhibitory output to underlying relay nuclei is required for the activation of low-voltage-gated T-type calcium channels and the subsequent burst firing of relay neurons, which is thought to be a contributing factor to SWD maintenance^15^. Thus, it has been argued that decreased RTN excitability is incompatible with SWD activity, pinpointing the probability of compensatory changes in the stargazer RTN^27^.

NMDAR activity and expression have been studied in pathological CT-TC circuit oscillation in genetic^42^,^43^,^44^,^45^,^46^ and pharmacological^47^ absence models, although no clear association has been found between altered NMDAR expression and abnormal CT-TC synchronicity. In contrast, pharmacological studies investigating the effect of NMDAR antagonists or agonists on SWD parameters have revealed contradicting results, in part, perhaps, due to disease heterogeneity and mechanistic differences in different animal models. Importantly, NMDAR antagonist treatments in stargazers *in vitro* and *in vivo* have suggested a mechanistic increase in NMDAR function in the RTN^27^, and pronounced influence to baseline interictal gamma power *in vivo*^37^. Notably, however, NMDAR antagonists bath applied to different nodes of the CT-TC circuit of stargazers *in vitro,* resulted in disparate outcomes. The competitive and non-competitive NMDAR blockers CPP and MK-801 led to exacerbation of cortical discharges in stargazer cortical slices^28^, whereas application of the competitive antagonist APV to thalamic preparations from stargazers decreased thalamic oscillatory activity^27^. The effects of i*n vivo* applied NMDAR antagonists on SWD activity in stargazers have also been ambiguous^28^,^48^, as one study demonstrated transient suppression of SWD activity upon the competitive antagonist CPP application, although this effect was superseded by a slight increase in SWD duration 20 min after injection^48^. In the same study, the non-competitive antagonist MK-801 led to decreased SWD duration, however, the authors noted the appearance of highly irregular EEG patterns following MK-801 injection^48^. In contrast, in a more recent study, MK-801 caused a marked increase in SWD duration 1 hour post-injection^28^, and also slowed the spike frequency from 6–9 Hz to 3–4 Hz^28^. This recent data suggest that NMDARs are indispensable for excitatory neurotransmission in the absence of AMPARs on inhibitory neurons in stargazers, and their blockade is likely to cause further reductions in inhibitory neuron excitability, and subsequent weakening of feed-forward inhibition of principal neurons, exacerbating SWDs^28^. Intriguingly, the baseline interictal relative gamma power was shown to be augmented in stargazers, which MK-801 was able to reverse, further indicating a profound role of NMDARs in the AMPAR-deficient stargazer PV^+^ cortical inhibitory interneurons^37^.

We hypothesised that the enhanced function in RTN is associated with increased protein expression levels. The role of NMDARs in the RTN is incompletely understood. Thorough electrophysiological characterization of NMDAR contribution at RTN synapses revealed a relatively weak NMDAR-component at corticothalamic (CT)-RTN^49^, but a more substantial contribution at thalamocortical (TC)-RTN synapses during regular synaptic transmission. In contrast, a recent study indicated a more significant NMDAR contribution at CT-RTN synapses relative to AMPARs^50^. Thalamic NMDARs were also shown to be required for the generation of thalamic spindle oscillations^51^. However, others demonstrated that the role of NMDARs in the CT-TC circuit is variable throughout postnatal development. NMDAR contribution is most prominent at early postnatal stages, but is less significant in the more developed circuit^52^,^53^. The developmental switch from NMDAR dominated ‘silent synapses’ to synapses predominated by AMPARs is maximal at corticothalamic and thalamocortical synapses of the RTN; while, strong contribution of NMDARs persists into adulthood in the VP thalamus^53^. Reports of dominant NMDAR function are also inconsistent with the expression data of ionotropic glutamate receptors in the RTN. AMPARs, particularly the GluA4 subunit, are prevalent in adult rodent RTN, especially compared to the VP^29^,^30^. Although the expression of NMDA receptors in rodent RTN has not been studied in as much detail, early work found that only about 30% of asymmetrical excitatory synapses in rat RTN immunolabelled with NMDAR1^54^. Also, NMDAR subunits in macaque^32^ and human^55^ RTN are expressed at very low levels in comparison to other thalamic nuclei. Our results are congruent with these data and indicate much lower NMDAR expression in the mouse RTN compared to VP. Functional tetrameric NMDARs are formed by two obligatory NR1 subunits combined with either NR2 or NR3 subunits, from the possible four NR2 (2A-D) or two NR3 (3A-B) gene products^56^,^57^,^58^. Our examination focused on the NR2A- and NR2B-containing NMDAR subtypes, because, although NMDAR functional properties were unaltered, changes in NMDAR conductance were most enhanced at depolarized membrane potentials in the stargazer RTN^27^. Expression of these subtypes, as well as total levels were, however, unchanged in the stargazer RTN. It would be interesting to examine NR2C-NMDARs, a prevalent subtype in the RTN^59^,^60^. However, the electrophysiological findings did not indicate large-scale remodelling of NMDAR composition or a specific increase of the NR2C subtype in stargazers relative to controls^27^, and the expression of functional receptors would ultimately be limited by the compulsory NR1 subunit. Similarly, potential changes to the expression of individual NR1 splice variants^61^ may contribute to functional differences in the stargazer RTN, but this was not investigated in this study. The NR1-specific antibody used recognizes all splice variants and, hence, encompasses all NMDA receptors, reflecting the total protein expression levels.

Importantly, NMDAR membrane redistribution could effectively counteract the effects of decreased AMPAR trafficking and stability, despite the lack of changes in tissue NMDAR expression. Indeed, NR1 protein expression was for example elevated at GC synapses in the stargazer cerebellum^19^, but NR subunit protein levels were found unchanged in stargazer cerebellar tissue with Western blot^62^. Hence, we evaluated synaptic NMDAR levels in stargazer thalamus, and, in particular, sought to correlate this with synaptic AMPARs levels. Unlike AMPARs, however, NMDARs were unchanged in the stargazer membrane fraction, suggesting that deficient AMPAR trafficking and synaptic anchorage is not directly associated with a ubiquitous increase in synaptic NMDAR levels. Nonetheless, previous findings demonstrated an extensive AMPAR loss specifically at the CT-RTN synapse in stargazers^25^, and a compensatory upregulation of NMDAR expression limited to this synapse could be concealed by pooling heterogeneous synaptic populations of the RTN and VP. Post-embedding immunogold-cytochemistry confirmed the significant decrease in GluA4 subunit at CT-RTN synapses in stargazers, signified by fewer GluA4 labelled PSD profiles and lower calculated mean gold particle numbers per labelled PSDs. On the other hand, no change could be detected in NR1 labelling in epileptic RTN single sections relative to non-epileptic sections.

NMDARs are relatively mobile and activity-dependent lateral diffusion between membrane compartments occurs on a time-scale of seconds to minutes in cell cultures^63^,^64^. Therefore, it could be that alterations in synaptic NMDARs are more dynamic and temporally closely associated with SWD activity and, therefore, could not be detected by the techniques we have utilized. On the other hand, findings regarding NMDAR mobility emerged from studies using cell cultures and contradict results from acute brain slices. Harris and Pettit (2007) could not demonstrate high NMDAR mobility in acute brain slices, but rather proved the existence of stable synaptic and extrasynaptic receptor pools with no mobility between compartments^65^, making it debatable how mobile NMDARs are *in vivo*. Moreover, the high rate of discharge frequency and the prolonged nature of discharges in stargazers^18^ suggest that, statistically, large proportions of thalamic synapses would be ‘captured’ at time points temporally corresponding to seizures. Because NMDAR function is heterogeneous in the RTN^49^,^66^, it is also possible that changes are limited to a subset of synapses in stargazer RTN. Overall, our findings so far suggest the lack of ubiquitous changes at thalamic synapses, and, in particular, at the CT-RTN synapse, however, this remains to be further investigated. Functional NMDAR changes could be mediated by different cellular mechanisms, such as posttranslational modification. Of note, expression of the full-length NR2A subunit was more variable in epileptic stargazer samples, which could be caused by proteolytic cleavage. Induced truncation of NR2A subunits has been observed in focal cerebral ischemia^67^ and is thought to be a regulatory mechanism for NMDAR localization and function in disorders^68^.

Changes to extrasynaptic glutamate receptor expression in stargazers may be accountable for differences in excitatory neurotransmission^27^, such as prolonged AMPAR-mediated transmission. In fact, we found measurable AMPAR expression, particularly GluA4, in the extrasynaptic plasma membrane fraction. In contrast to increased perisynaptic GluA4 density shown by array tomography^27^, there was no statistically significant change in the amount of extrasynaptic GluA subunits in the epileptic stargazers relative to control extrasynaptic samples in this study. Nonetheless, this still entails elevated extrasynaptic to synaptic ratio for GluA4 subunits in the epileptic stargazers compared to controls. On the other hand, we were unable to quantify the expression of extrasynaptic NMDARs, and no apparent increase in the proportion of extrasynaptic NMDARs in the stargazer thalamus could be detected. Membrane-associated GluA4 and NR1 immunogold labelling was detectable away from distinct PSD profiles with post-embedding cytochemistry, however, the post-embedding immunolabelling was performed on single sections and synaptic profiles were not reconstructed using serial sectioning, thus, definitive categorization of these particles into membrane domains would be difficult^69^,^70^.

Altogether, enhanced NMDAR-mediated currents are unlikely to be mediated by increased translation or stability of NMDAR subunits. The findings also provide some evidence to suggest that synaptic NMDAR expression is unchanged in the stargazer RTN and VP, and at the CT-RTN synapse. Therefore, it remains unresolved if RTN neurons can be efficiently recruited via the corticothalamic input in the stargazer model. In light of the dramatic reduction in AMPAR expression and function, it continues to be highly likely that RTN neurons are hypoexcited upon CT input, but efficiently excited by TC projections. The ensuing diminished feed-forward inhibition of VP may lead to hyperexcited VP neurons, which can result in enhanced feedback inhibition onto VP neurons, following the thalamocortical recruitment of RTN neurons. The secondary changes observed in stargazers, in particular enhanced tonic inhibition^71^, and increased expression of GABAR subunits in VP^72^,^73^, are compatible with this model, as these may be compensatory mechanisms to overcome the weakened feed-forward inhibitory input.

Absence epilepsy is a heterogeneous disease, which is also reflected by the varied response of patients to available anti-epileptic drugs (AED). Since AEDs can lead to paradoxical, proepileptic response^28^,^74^ in some patients, it is critical to have a full understanding of cellular changes in the CT-TC circuit, as these undetected changes could underlie the proepileptic response to AEDs. Detailed understanding of anatomical and functional changes will also help develop patient-specific treatment strategies. Our results contribute to the elucidation of anatomical and molecular changes in the pathological CT-TC circuit.

## Methods

### Animals

All experiments were performed on adult (8–12 weeks) male stargazer (stg/stg) and control (+/stg, +/+) littermate mice. Mice were bred and housed at University of Otago Animal Facilities from breeding stock obtained from Jackson Laboratory (Bar Harbor, ME, USA). To verify our colony genotyping for the stargazin gene was performed, using the following primers (Jackson Lab): TAC TTC ATC CGC CAT CCT TC forward; TGG CTT TCA CTG TCT GTT GC reverse for wild-type allele; GAG CAA GCA GGT TTC AGG C mutant allele reverse (Fig. 1). Western blot was performed to confirm the effect of the mutation at the protein level. All animal procedures were approved by the University of Otago Animal Welfare and Ethics Committee and were performed in accordance with University guidelines and regulations.

### Brain microdissection

Adult male stargazer and control littermates were sacrificed by means of cervical dislocation, brains were carefully removed and snap frozen on dry ice. Frozen brains were sectioned in a freezing cryostat into 250 μm thick coronal sections, which were immediately thaw-mounted on glass slides, then re-frozen. Sections containing the reticular thalamic nucleus (RTN) and underlying ventral posterior (VP) relay nucleus were identified and the nuclei located based on anatomical landmarks. For the subsequent studies, RTN and VP were either harvested separately or pooled together. For the nucleus-specific microdissection^75^,^76^, 24 G blunted needles, with an inner diameter of 300 μm, were used to extract the somatosensory sectors of RTN (Fig. 2a) and VP (Fig. 3a), bilaterally. Sample purity was also assessed using markers in the subsequent Western blot analysis: VGlut1 (Synaptic Systems, 135 311) and GluA4 (Millipore, AB1508), which have higher expression levels in VP and RTN, respectively.

For the synapse-specific study, synaptic membrane fraction was biochemically isolated^36^ from tissue, collected with biopsy punch pens of 1 mm (Miltex), medial and adjacent to the internal capsule (Fig. 4a).

### Western blot of RTN and VP

Micropunches of RTN and VP were homogenized by sonication in 50 mM Tris, 2 mM EDTA, 3% SDS, pH6.8 (supplemented freshly with PMSF and protease inhibitor cocktail Sigma, P8340) buffer. Sample protein concentration was quantified using DC Protein assay (Bio-Rad, 500–0116), and 15 μg protein per sample, denatured by β-mercaptoethanol was separated on SDS-PAGE gels. Separated proteins were transferred onto nitrocellulose membranes, which were then subjected to immunoblot analysis. Importantly, samples from non-epileptic (NE) and epileptic (E) littermates, as well as samples from RTN and VP from littermates were processed parallel, transferred on to the same membrane for comparative analysis. Expression of AMPAR subunit GluA4 (Millipore, AB1508) and NMDAR subunits NR1 (Millipore, MAB1586), NR2A (Millipore, AB1555P), and NR2B (Millipore, AB1557P) was probed by IR fluorescence immunoblot (Odyssey, LI-COR Biosciences) using previously characterised antibodies. Details and references regarding antibodies used in this study can be found on respective company websites. To allow the simultaneous probing of target AMPA- and NMDAR subunit proteins, and regional and loading controls, blots were cut horizontally. Membranes after transfer were washed in Tris-buffered saline (TBS), blocked in Odyssey Blocking Buffer (OBB) or Casein Blocking Buffer (LI-COR Biosciences) for 30–45 minutes at room temperature and incubated overnight with the antibodies above diluted in OBB diluted 2-fold with TBS-0.1%Tween. Following primary antibody incubation membranes were extensively washed with TBS-Tween before being incubated with IRDye-coupled anti-rabbit and anti-mouse secondary antibodies (LI-COR Biosciences) diluted 1:10000 in TBS-Tween for 1 hour at room temperature. Membranes were then washed in TBS-Tween and TBS before being scanned on Odyssey Classic imaging platform (LI-COR Biosciences). Image Studio^TM^ was utilized for quantification by measuring integrated intensity values of bands corresponding to the respective receptor subunit molecules. All values were normalized against β-actin (Abcam, ab8226 or 8227). Expression in epileptic (E) mice is expressed in relation to NE (E/NE). Comparative analysis was performed on samples processed in parallel, and imaged using the same equipment and software settings.

### Biochemical fractionation and Western blot of thalamic biopsy punches

Bilateral thalamic punches obtained from frozen coronal brain sections by biopsy punch pens of 1mm (Miltex) were homogenized in ice-cold fractionation buffer of 10 mM Tris, pH7.4 containing 1 mM EDTA and 320 mM sucrose, and supplemented fresh with PMSF and protease inhibitor cocktail (P8340, Sigma) by using sterile plastic pestles and pulses of ultrasound. To isolate synaptic membranes, a multistep centrifugation protocol was used as of Davies *et al*.^36^ (Fig. 4b). In brief, homogenates were first spun at 1000 g for 10 minutes to pellet debris and nuclei. The supernatant of the first spin was centrifuged at 10000 g for 15 mins, pelleting the cell membrane. This fraction was re-suspended in 0.5% Triton X-100-containing fractionation buffer and incubated on ice for 40 minutes before being spun at 32000 g for 20 minutes. This step enables the separation of the Triton X-100-insoluble synaptic membrane (pellet) from extrasynaptic membranes (supernatant). The pellet was re-suspended in 50 mM Tris, 2 mM EDTA, 3% SDS, pH6.8 buffer, while the supernatant was first concentrated by acetone, followed by centrifugation and re-suspension in the Tris/EDTA/SDS buffer for Western blot (WB). 10 μg protein of every fraction was subsequently processed by IR fluorescence WB in a series of experiments. WB methodology was as of above. Antibodies used, apart from listed ones above, were NR2B (610416, BD Transduction), GluA2 (Millipore, MAB397), Pan-Cadherin (4068, Cell Signaling Technology), PSD95 (Synaptic Systems, 124 011) and synaptophysin (Synaptic Systems, 101 004). Cadherin was used as internal loading control in subsequent data analysis due to the lack of direct association between cadherins and TARPs^24^.

### Post-embedding immunogold cytochemistry – electron microscopy

Tissue processing and EM immunocytochemistry were performed following previously established methods^25^,^73^,^77^. Adult male stargazer and control mice from three separate litters were terminally anesthetized, and transcardially perfused with 4% paraformaldehyde-0.1% glutaraldehyde in 0.1 M PB, pH7.4. Brains were further immersion fixed overnight at 4 °C, subsequently rinsed in 0.1 M PB and 250 μm thick coronal sections were cut on a vibratome (Vibratome^®^ 1500, The Vibratome Company). Tissue blocks containing the internal capsule and RTN were trimmed and cryprotected through slow infiltration in sucrose solutions of increasing concentrations. Cryoprotected tissue was slam-frozen (Leica KF80, Leica Microsystems), freeze-substituted in methanol and embedded into Lowicryl HM-20 (14340, EMS) in a Leica automatic freeze-substitution system (AFS, Leica). Semi- and subsequently ultra-thin (70–80 nm) sections were cut and mounted on formvar-coated nickel grids for immunolabelling. Grids were incubated on droplets of primary antibody solutions diluted in 5 mM Tris – 0.7% NaCl, pH7.6 (AMPAR-GluA4 Millipore AB1508^25^; NMDAR-NR1 BD Pharmingen 556308^78^), followed by incubation in goat anti-rabbit and goat anti-mouse IgGs coupled to 15 and 10 nm gold, respectively (EM.GAR15 and EM.GFAM10; BBI), diluted in 5 mM Tris – 0.7% NaCl, pH8.2. Primary antibody omission controls were included in each experiment. After light counterstaining with uranyl acetate and lead citrate grids were examined under Phillips CM100 transmission electron microscope (Phillips/FEI Corporation) fitted with MegaView III digital camera (Olympus Soft Imaging Solutions GmbH). Stargazer and control tissue were processed in parallel, and the experimenter was blinded to avoid bias. Images were analysed in Fiji (Fiji Is Just ImageJ) open source software. Gold particles that were within 30 nm from the midline of labelled PSDs were considered to represent synaptic expression and were counted to determine synaptic GluA4 and NR1 levels. The proportion of labelled versus unlabelled PSDs was determined. Individual PSD-associated gold particles for NE and E experimental groups were combined for statistical analysis^38^.

### Analysis and figure preparation

Scatter plots combined with bars are used to display individual values as well as mean ± SEM. Statistical analysis was performed in Prism 6, using one sample t-test to probe statistical significance for relative E expression in WB experiments and unpaired two-sample t-test with Welch’s correction in EM immunogold experiments. Huber-White robust error analysis was performed in Stata to test inter-litter heteroscedasticity in NMDAR subunit expression in the regional and fractionation WB experiments. Composite Western blot images were prepared, using cropped images, in Photoshop CS6. Brightness and contrast were only adjusted slightly, and equally across blots, including control and epileptic samples. Figures were compiled in Photoshop CS6.

## Additional Information

**How to cite this article:** Barad, Z. *et al*. NMDA Receptor Expression in the Thalamus of the Stargazer Model of Absence Epilepsy. *Sci. Rep.* **7**, 42926; doi: 10.1038/srep42926 (2017).

**Publisher's note:** Springer Nature remains neutral with regard to jurisdictional claims in published maps and institutional affiliations.
